# Step-Based Dosing of Anticoagulants in COVID-19 Treatment

**DOI:** 10.7759/cureus.67256

**Published:** 2024-08-19

**Authors:** Minh-Hoang Tran, Hoang Hai Nguyen, Quang Trung Nguyen, Thy Doan Minh Tran, Kim-Huong Truong-Nguyen, Hong Tham Pham

**Affiliations:** 1 Therapeutics, NTT Hi-Tech Institute, Nguyen Tat Thanh University, Ho Chi Minh City, VNM; 2 Cardiology, Nhan Dan Gia Dinh Hospital, Ho Chi Minh City, VNM; 3 Pharmacy, Nhan Dan Gia Dinh Hospital, Ho Chi Minh City, VNM

**Keywords:** vietnam, venous thromboembolism (vte), bleeding, mortality, anticoagulants, covid-19

## Abstract

Background: Step-based dosing of anticoagulants has been widely implemented for the treatment of coronavirus disease 2019 (COVID-19), but no studies have comprehensively evaluated the effectiveness and safety of this approach. We aimed to investigate whether step-based dosing of anticoagulants was associated with clinical outcomes in patients with COVID-19 compared with standard prophylactic dosing.

Method: We conducted a retrospective cohort study on adults hospitalized with moderate-to-severe COVID-19. The exposure was step-based dosing of anticoagulants, including prophylactic anticoagulants (PrA), prophylactic-switching-to-therapeutic anticoagulants (Pr-to-ThA), therapeutic anticoagulants (ThA), and therapeutic-switching-to-prophylactic anticoagulants (Th-to-PrA). The primary effectiveness outcome was a composite of all-cause mortality, admission to an intensive care unit (ICU admission), stroke, and venous thromboembolism (VTE). The primary safety outcome was a composite of major and minor/clinically relevant non-major (CRNM) bleeding.

Results: Among 1,081 records for analysis (mean age 59.9, 49.9% being female), during a median follow-up of 15 days, the primary effectiveness outcome occurred in 333 patients (33.5% in the PrA group, 24.6% in the Pr-to-ThA group, 23.7% in the Th-to-PrA group, and 38.0% in the ThA group). Compared with the PrA group, patients receiving Pr-to-ThA had a lower risk of the primary effectiveness outcome (adjusted odds ratio (OR) 0.64, 95% CI: 0.45 to 0.90, Dunnett-adjusted p = 0.01), while those in the Th-to-PrA and ThA were more likely to experience the primary safety outcome (Th-to-PrA, aOR = 3.00, 95% CI: 1.53 to 5.89; ThA, aOR = 3.05, 95% CI: 1.61 to 5.79).

Conclusion: In adults hospitalized with moderate-to-severe COVID-19, compared with standard PrA, the step-based dose-increasing therapy was associated with a lower composite risk of all-cause mortality, ICU admission, stroke, or VTE without evidence of a higher risk of bleeding. ThA dosing was associated with an increase in the bleeding risk, primarily minor and CRNM bleeding.

## Introduction

Anticoagulants have been endorsed globally as the essential therapy for the treatment of hospitalized patients with coronavirus disease 2019 (COVID-19) [1, 2]. However, the optimal dosing strategy for anticoagulants can vary among different clinical settings [1-4], as comprehensive and robust evidence is still lacking. Many guidelines recommend a fixed-dose approach for patients with moderate-to-severe COVID-19 [1-4], which might not be the best solution in clinical practice. This is probably because the clinical status and biomarkers of hospitalized patients could change rapidly and unexpectedly [5], unlike the recommendations of COVID-19 guidelines.

To overcome this issue, some Asian countries implemented a step-based approach to anticoagulant therapy. In this approach, anticoagulant dosing depended on the risk of venous thromboembolism (VTE), which was assessed using C-reactive protein, D-dimer, ferritin, interleukin-6, and pulmonary infiltrates [6]. Current evidence has not covered this strategy, as most clinical trials only focused on the fixed dose of anticoagulants [7-11]. A major challenge when investigating the step-based approach was disentangling the effects of different doses of anticoagulants from escalating/de-escalating practices. This lack of evidence could limit the applicability of the step-based approach, especially in patients with unstable hemodynamics. In these cases, the fixed-dose approach might either increase the risk of VTE or bleeding.

To address this evidence gap, we aimed to investigate whether step-based dosing of anticoagulants was associated with improved outcomes in patients with moderate-to-severe COVID-19 compared with the standard prophylactic dosing. In addition, we also aimed to disentangle the associations of the initial and final dosing from the overall estimates to identify if increasing or decreasing anticoagulant doses were associated with any benefits.

## Materials and methods

Design, setting, and participants

We conducted a retrospective cohort study at Nhan Dan Gia Dinh (NDGD) Hospital, a 1,500-bed general Vietnamese hospital in Ho Chi Minh City, Vietnam, that has provided COVID-19 treatment since the first outbreak. We screened and included the medical records of patients who: (1) were ≥18 years old; (2) got confirmed COVID-19 (the COVID-19 diagnosis was verified by either a positive rapid antigen test with typical symptoms or a positive real-time polymerase chain reaction test); (3) were hospitalized at NDGD Hospital during the period between August 1, 2021, and November 30, 2021; (4) had moderate-to-severe illness at admission (Appendix A); and (5) received at least one dose of heparin-based anticoagulants. The records were excluded if the patients: (1) were pregnant or breastfeeding; (2) were moderately or severely immunocompromised (Appendix A); (3) had renal impairment (creatinine clearance, CrCl, <30 mL/minute); (4) had hepatic impairment (Child-Pugh class C); or (5) required therapeutic anticoagulation or dual antiplatelet therapy for underlying conditions. The follow-up period was from hospitalization to discharge (either as completing the treatment, transferring to another healthcare institution, or deceased).

This study was approved by the ethics committee of NDGD Hospital, Ho Chi Minh City, Vietnam, under approval number 62-2021/NDGD-HDDD. We only used retrospective data from health records while strictly maintaining patient confidentiality. Therefore, the Ethics Committee of NDGD Hospital stated that no separate informed consent was required for this study, except for the consent to data sharing for research purposes upon hospitalization. We reported this study in accordance with the Strengthening the Reporting of Observational Studies in Epidemiology (STROBE) Statement (Appendix B).

Exposure

The exposure of interest in this study was the clinical step-based dosing of anticoagulants. This divided the study sample into four cohorts, of which patients were given: (1) prophylactic anticoagulants (PrA); (2) prophylactic and later switching to therapeutic anticoagulants (Pr-to-ThA); (3) therapeutic anticoagulants (ThA); and (4) therapeutic and later switching to prophylactic anticoagulants (Th-to-PrA). The timing for switching in the Pr-to-ThA and Th-to-PrA groups could be anytime between day 1 (first dose) and day 10. If there was a switch after day 10, patients were classified based only on their first doses of anticoagulants, which were either PrA or ThA. Detailed definitions of each cohort are listed in Appendix A.

The dosing strategies of anticoagulants were decided depending on the risk of VTE, which followed a step-based national protocol (Appendix A). In our study setting, as new evidence of VTE in COVID-19 emerged rapidly, different physicians had different approaches for dosing anticoagulants. During the COVID-19 outbreaks, adherence to the unofficial guidance was not strictly imposed at our hospital. Thus, many physicians did not comply with the national protocol, resulting in some variations of the dosing strategies. Therefore, the lack of counterfactual outcomes and covariate overlap were unlikely to happen in our study. All patients received the guideline-recommended standard of care [1,2,6,12].

Outcomes

The primary effectiveness outcome was a composite of all-cause mortality, admission to an intensive care unit (ICU admission), stroke (either ischemic or hemorrhagic), and venous thromboembolism (VTE). The secondary effectiveness outcomes included individual components of the primary effectiveness outcome and mechanical ventilation. The primary safety outcome was a composite of major and minor/clinically relevant non-major (CRNM) bleeding, defined using the classification of the International Society on Thrombosis and Haemostasis [13,14]. The secondary safety outcomes were the two components of the primary safety outcome. The net-benefit outcome was a composite of all-cause mortality, ICU admission, stroke, VTE, and major bleeding. To avoid ascertainment bias, outcome measurement was conducted independently by the hospital staff under the supervision of the adjudication committee.

Covariates

We included the following covariates as potential prognostic factors: age (years, <65 or ≥65), sex (female or male), body mass index (kg/m^2^), chronic comorbidities (cardiovascular diseases, endocrine diseases, respiratory diseases, gastrointestinal diseases, or cancers), current smoker (yes or no), vaccination status (fully, partially, or no vaccination), biomarkers for treatment (C-reactive protein, D-dimer, and ferritin), and concurrent medications for COVID-19 treatment (corticosteroids, antivirals, antibiotics/antifungals). We did not control for variables that could be on the causal pathways from the exposure to the outcomes, as that may overadjust the estimated effects of anticoagulants on the outcomes.

Statistical analysis

We screened all medical records for inclusion (n = 1136) and included all eligible ones for analysis (n = 1081). We presented categorical variables as frequency with percentage and continuous variables as mean with standard deviation (SD) if normally distributed or median with interquartile range (IQR) if non-normally distributed. In our study setting, there was only a small rate of incomplete or missing data. Thus, we only used the complete-case analysis to investigate the association between the exposure and outcomes.

In the primary analysis, all primary and secondary outcomes were analyzed using g-estimation with bootstrapping to address the time-varying covariates. We used odds ratio (OR) with the 95% confidence interval (95% CI) to report the associations between the exposure and the outcomes. The reference level of the exposure for analysis was PrA. All the assumptions of g-estimation were justified based on our study design and the chronological order of data generation. We only conducted two-sided hypothesis testing for the primary effectiveness outcome, for which we used the Dunnett test to control for multiplicity with a family-wise error rate of 5%. All other outcomes were considered exploratory. We decided, a priori, to conduct a subgroup analysis on the primary effectiveness outcome for the cohorts with significant differences (Dunnett-adjusted p<0.05). The following subgroups were pre-specified: age (<65 (non-elderly) or ≥65 (elderly)), sex (female or male), chronic comorbidities (cardiovascular diseases, endocrine diseases, gastrointestinal diseases, or cancers), and vaccination status (≥1 dose or no dose). As this was for exploratory purposes, we did not control for multiplicity. To address the concerns about reverse causation and immortal time bias, we also conducted a landmark analysis where time 0 was set at day 10 after hospitalization. We excluded patients with outcomes occurring before this time from the landmark analysis. The modified follow-up period was from day 10 to discharge.

In the dose-switching cohorts, the overall associations could include the partial associations from the initial and switching doses of anticoagulants. To disentangle these, we used an exploratory model-based causal mediation analysis with 1,000 simulations based on the quasi-Bayesian Monte Carlo method [15,16]. The Probit link was used to model the outcomes using logistic regression. We set an interaction term between the dose strategies (prophylactic or therapeutic) of anticoagulants at days one and 10. The estimates were reported on a probability scale with a 95% CI. Negative signs implicated reversed associations of the exposure on the outcomes. The assumption of sequential ignorability for the causal mediation analysis was investigated by conducting a sensitivity analysis to check for the possible existence of unobserved pre-treatment covariates. In this analysis, a correlation parameter ≥0.4 was required to reverse the associations of the dose-switching strategies, which was large enough to confirm the robustness of the results in case the assumption was violated.

We conducted all statistical analyses using R software (version 4.2.1, R Foundation for Statistical Computing, Vienna, Austria). Apart from the base package, we also used the lavaan package for g-estimation, multcomp package for the Dunnett test, and mediation package for the exploratory causal mediation analysis [16-18].

## Results

Baseline characteristics

**Figure 1:**
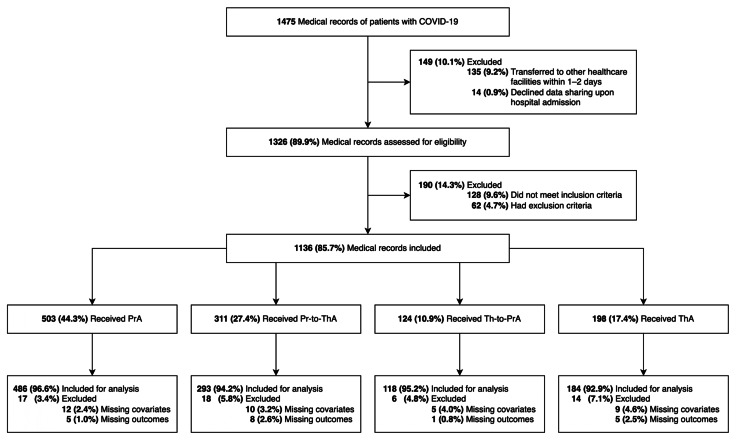
Flowchart of the study COVID-19: coronavirus disease 2019; PrA: prophylactic anticoagulants; Pr-to-ThA: prophylactic and switching to therapeutic anticoagulants; Th-to-PrA: therapeutic and switching to prophylactic anticoagulants; ThA: therapeutic anticoagulants

Patient characteristics of the included records for analysis (486 in the PrA group, 293 in the Pr-to-ThA group, 118 in the Th-to-PrA group, and 184 in the ThA group, Figure 1) were comparable to that of those being excluded (Appendix C). Among 1,081 records (all patients being Vietnamese-origin Asians), the mean age was 59.9 (SD 15.1), with about half of the sample being female (49.9%) or overweight/obese (51.0%). Over 68% of the patients were partially or fully vaccinated before hospitalization, but only 5% received a booster dose. The median times for dose switching in the Pr-to-ThA and Th-to-PrA groups were seven days and six days, respectively. Further details of the exposure-stratified characteristics are summarized in Table 1.

**Table 1:** Baseline characteristics. BMI: body mass index; CRP: C-reactive protein; IQR: interquartile range; PrA: prophylactic anticoagulants; Pr-to-ThA: prophylactic and switching to therapeutic anticoagulants; SD: standard deviation; Th-to-PrA: therapeutic and switching to prophylactic anticoagulants; ThA: therapeutic anticoagulants ^a^ Percentages may not equate to 100 due to concurrent status or rounding. ^b^ Current smoker refers to an adult who has smoked ≥100 cigarettes in their lifetime and who currently smokes cigarettes. ^c^ Fully vaccinated persons were those who received at least two doses (either homologous or heterologous) of the approved vaccines for at least 2 weeks before getting the first COVID-19. Partially vaccinated persons were those who received only 1 dose before getting the first COVID-19. The two approved vaccines in Vietnam included BNT162b2 (Pfizer/BioNTech), mRNA-1273 (Moderna), AZD1222 (AstraZeneca), and BBIBP-CorV (Sinopharm). ^d^ Corticosteroids included dexamethasone, methylprednisolone, and prednisolone. ^e^ Antivirals for COVID-19 treatment included remdesivir, molnupiravir, and favipiravir. ^f^ Antibiotics or antifungals were prescribed for patients with confirmed secondary infections. Treatments with these medications could be empirical and/or based on the susceptibility of the isolated pathogens.

Characteristics	PrA (n = 486)	Pr-to-ThA (n = 293)	Th-to-PrA (n = 118)	ThA (n = 184)	Total (n = 1081)
Age (years), mean ± SD	60.3 ± 15.1	59.9 ± 15.5	60.0 ± 14.5	58.8 ± 14.5	59.9 ± 15.1
Female sex, n (%)	254 (52.3)	144 (49.1)	46 (39.0)	95 (51.6)	539 (49.9)
BMI (kg/m^2^), mean ± SD	23.4 ± 4.6	23.5 ± 4.4	24.4 ± 4.6	24.5 ± 4.5	23.7 ± 4.5
Chronic comorbidities, n (%)^a^					
Cancers	31 (6.4)	22 (7.5)	3 (2.5)	7 (3.8)	63 (5.8)
Cardiovascular diseases	230 (47.3)	137 (46.8)	70 (59.3)	103 (56.0)	540 (50.0)
Endocrine diseases	159 (32.7)	99 (33.8)	53 (44.9)	80 (43.5)	391 (36.2)
Gastrointestinal diseases	36 (7.4)	23 (7.8)	17 (14.4)	25 (13.6)	101 (9.3)
Respiratory diseases	32 (6.6)	16 (5.5)	8 (6.8)	11 (6.0)	67 (6.2)
Current smoker, n (%)^b^	72 (14.8)	55 (18.8)	20 (16.9)	32 (17.4)	179 (16.6)
Vaccination status, n (%)^a,c^					
Fully	188 (38.7)	115 (39.2)	37 (31.4)	61 (33.2)	401 (37.1)
Partially	147 (30.2)	98 (33.4)	34 (28.8)	58 (31.5)	337 (31.2)
None	151 (31.1)	80 (27.3)	47 (39.8)	65 (35.3)	343 (31.7)
Biomarkers, median (IQR)					
CRP (mg/L)	11.4 (6.3–13.7)	10.9 (6.8–12.1)	71.2 (44.5–92.1)	77.8 (32.9–118.4)	52.5 (13.7–84.4)
D-dimer (ng/mL)	1325 (1184–1790)	1547 (1338–1859)	2162 (1581–2736)	2377 (1613–2710)	1448 (1359–1805)
Ferritin (ng/mL)	682 (536–870)	588 (479–812)	1268 (692–1830)	1356 (775–2025)	637 (362–1253)
Concurrent medications, n (%)^a^					
Corticosteroids^d^	470 (96.7)	284 (96.9)	105 (89.0)	176 (95.7)	1035 (95.7)
Antivirals^e^	105 (21.6)	82 (28.0)	36 (30.5)	64 (34.8)	287 (26.5)
Antibiotics/antifungals^f^	146 (30.0)	75 (25.6)	85 (72.0)	164 (89.1)	470 (43.5)

Primary outcomes

**Figure 2:**
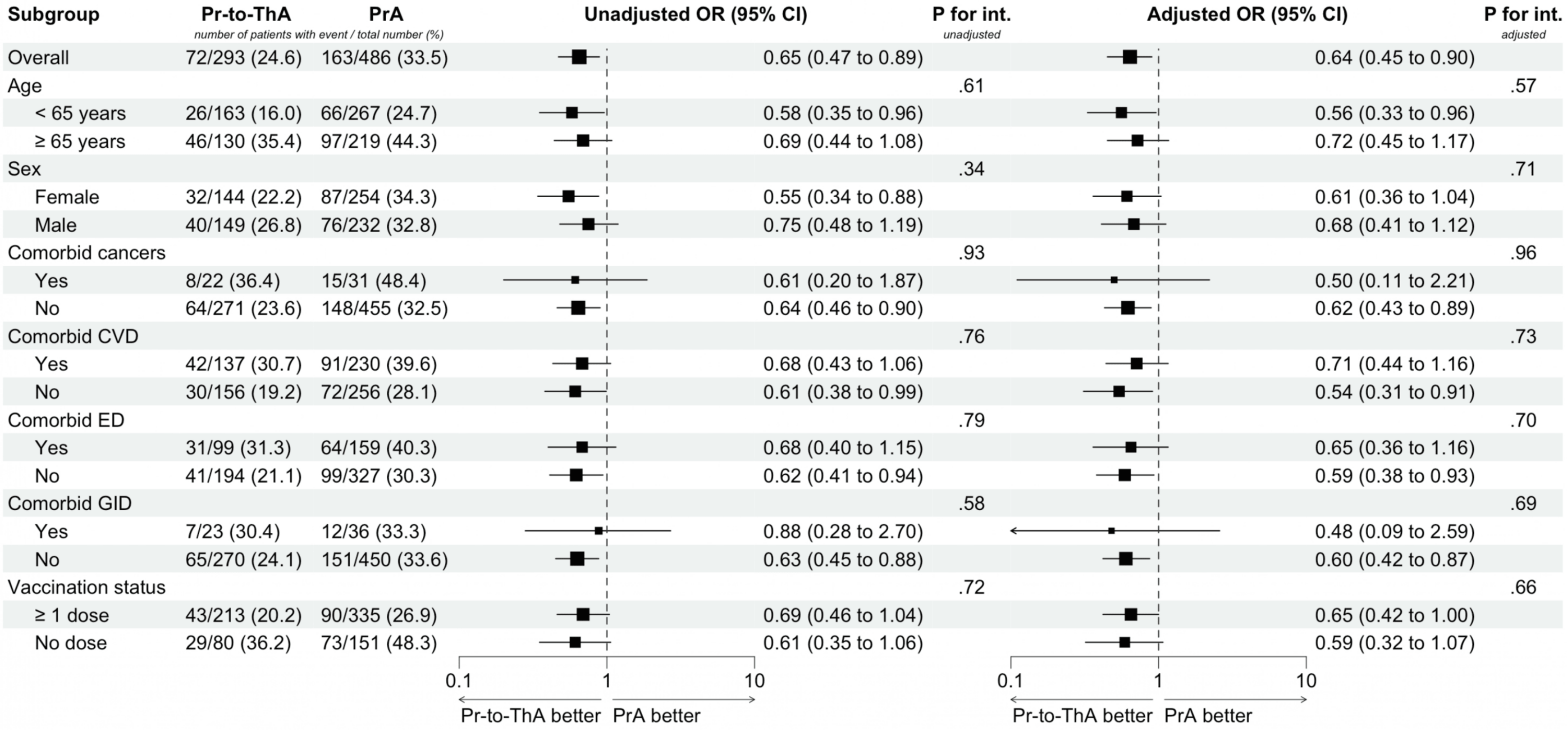
Subgroup analysis between dose-increasing and standard prophylactic anticoagulants CI: confidence interval; CVD: cardiovascular diseases; ED: endocrine diseases; GID: gastrointestinal diseases; OR: odds ratio; P for int: p-value for interaction; PrA: prophylactic anticoagulants; Pr-to-ThA: prophylactic and switching to therapeutic anticoagulants Adjusted ORs were controlled for age (years), sex (female or male), body mass index (kg/m^2^), chronic comorbidities (cardiovascular diseases, endocrine diseases, respiratory diseases, gastrointestinal diseases, or cancers), current smoker (yes or no), vaccination status (fully, partial, or no vaccination), biomarkers for treatment (CRP, D-dimer, and ferritin), and concurrent medications for COVID-19 treatment (corticosteroids, antivirals, antibiotics/antifungals). For each subgroup being investigated, we excluded the corresponding variable from the model specification of g-estimation.

During a median follow-up of 15 days (IQR 12-18), the primary effectiveness outcome occurred in 333 out of 1,081 patients (163/486 (33.5%) in the PrA group, 72/293 (24.6%) in the Pr-to-ThA group, 28/118 (23.7%) in the Th-to-PrA group, and 70/184 (38.0%) in the ThA group (Table 2). Compared with the PrA group, patients receiving Pr-to-ThA and Th-to-PrA had lower risks of developing the primary effectiveness outcome (Pr-to-ThA: adjusted OR=0.64, 95% CI: 0.45 to 0.90, original p=0.01; Th-to-PrA: adjusted OR=0.57, 95% CI: 0.34 to 0.95, original p=0.03, Table 2). After controlling for multiplicity, only the association in patients receiving Pr-to-ThA was significant (Dunnett-adjusted p = 0.03, Table 2). Results of the landmark analysis also supported these findings (Table 3). There was no difference in the subgroups between patients on Pr-to-ThA and PrA therapies (all p for interaction >0.05, Figure 2). Associations between the covariates and the primary effectiveness outcomes are listed in Appendix D.

**Table 2:** Overall associations between dosing of anticoagulants and the outcome CRNM: clinically relevant non-major; ICU: intensive care unit; IQR: interquartile range; OR: odds ratio; PrA: prophylactic anticoagulants; Pr-to-ThA: prophylactic and switching to therapeutic anticoagulants; Th-to-PrA: therapeutic and switching to prophylactic anticoagulants; ThA: therapeutic anticoagulants; VTE: venous thromboembolism ^a^ PrA was the reference level for all statistical comparisons. ^b^ ORs were presented with a 95% confidence interval, which were calculated using g-estimation with bootstrapping. ^c^ Adjusted OR were controlled for age (years), sex (female or male), body mass index (kg/m2), chronic comorbidities (cardiovascular diseases, endocrine diseases, respiratory diseases, gastrointestinal diseases, or cancers), current smoker (yes or no), vaccination status (fully, partially, or no vaccination), biomarkers for treatment (CRP, D-dimer, and ferritin), and concurrent medications for COVID-19 treatment (corticosteroids, antivirals, antibiotics/antifungals). ^d^ The primary effectiveness outcome was a composite of all-cause mortality, ICU admission, stroke (either ischemic or hemorrhagic), and VTE. ^e^ Original p=0.01 and adjusted p=0.03 (Dunnett test, family-wise error rate of 5%). ^f^ Original p=0.03 and adjusted p=0.09 (Dunnett test, family-wise error rate of 5%). ^g^ Original p=0.23 and adjusted p=0.53 (Dunnett test, family-wise error rate of 5%). ^h^ These outcomes were not adjusted for multiplicity and should be interpreted as exploratory. ^i^ These estimates were unreliable due to too few events being observed. ^j^ The primary safety outcome was a composite of major and minor/CRNM bleeding, defined using the classification of the International Society on Thrombosis and Haemostasis. ^k^ The net-benefit outcome was a composite of all-cause mortality, ICU admission, stroke, VTE, and major bleeding.

Outcomes	PrA^a^ ( n= 486)	Pr-to-ThA (n = 293)			Th-to-PrA (n = 118)			ThA (n = 184)		
	n (%)	n (%)	Unadjusted OR^b^	Adjusted OR^b,c^	n (%)	Unadjusted OR^b^	Adjusted OR^b,c^	n	Unadjusted OR^b^	Adjusted OR^b,c^
Primary effectiveness outcome^d^	163 (33.5)	72 (24.6)	0.65 (0.47 to 0.89)	0.64 (0.45 to 0.90)^e^	28 (23.7)	0.62 (0.39 to 0.98)	0.57 (0.34 to 0.95)^f^	70 (38.0)	1.22 (0.86 to 1.73)	1.30 (0.85 to 1.99)^g^
Secondary effectiveness outcomes^h^										
All-cause mortality	117 (24.1)	53 (18.1)	0.70 (0.48 to 1.00)	0.71 (0.48 to 1.05)	16 (13.6)	0.49 (0.28 to 0.87)	0.43 (0.23 to 0.82)	51 (27.7)	1.21 (0.82 to 1.78)	1.43 (0.88 to 2.32)
ICU admission	157 (32.3)	71 (24.2)	0.67 (0.48 to 0.93)	0.67 (0.47 to 0.95)	24 (20.3)	0.54 (0.33 to 0.87)	0.49 (0.28 to 0.84)	40 (21.7)	0.58 (0.39 to 0.87)	0.59 (0.37 to 0.94)
Stroke	1 (0.2)	1 (0.3)	– ^i^	– ^i^	0 (0.0)	– ^i^	– ^i^	1 (0.5)	– ^i^	– ^i^
VTE	29 (6.0)	16 (5.5)	0.91 (0.49 to 1.71)	0.89 (0.47 to 1.70)	6 (5.1)	0.84 (0.34 to 2.08)	0.78 (0.29 to 2.07)	9 (4.9)	0.81 (0.38 to 1.75)	0.69 (0.29 to 1.64)^e^
Mechanical ventilation	76 (15.6)	43 (14.7)	0.93 (0.62 to 1.39)	0.93 (0.60 to 1.43)	21 (17.8)	1.17 (0.69 to 1.99)	1.56 (0.85 to 2.85)	23 (12.5)	0.77 (0.47 to 1.27)	1.01 (0.55 to 1.83)^e^
Primary safety outcome^h,j^	35 (7.2)	22 (7.5)	1.05 (0.60 to 1.82)	1.05 (0.60 to 1.86)	19 (16.1)	2.47 (1.36 to 4.50)	3.00 (1.53 to 5.89)	28 (15.2)	2.31 (1.36 to 3.93)	3.05 (1.61 to 5.79)^e^
Secondary safety outcomes^h^										
Major bleeding	5 (1.0)	4 (1.4)	– ^i^	– ^i^	2 (1.7)	– ^i^	– ^i^	4 (2.2)	– ^i^	– ^i^
Minor/CRNM bleeding	30 (6.2)	18 (6.1)	0.99 (0.54 to 1.82)	1.00 (0.54 to 1.85)	17 (14.4)	2.56 (1.36 to 4.82)	2.89 (1.43 to 5.85)	24 (13.0)	2.28 (1.29 to 4.02)	2.83 (1.44 to 5.55)
Net-benefit outcome^h,k^	166 (34.2)	75 (25.6)	0.66 (0.48 to 0.92)	0.65 (0.46 to 0.92)	29 (24.6)	0.63 (0.40 to 0.99)	0.60 (0.36 to 1.00)	73 (39.7)	1.27 (0.89 to 1.80)	1.41 (0.92 to 2.16)

**Table 3:** Landmark analysis CRNM: clinically relevant non-major; ICU: intensive care unit; IQR: interquartile range; OR: odds ratio; PrA: prophylactic anticoagulants; Pr-to-ThA: prophylactic and switching to therapeutic anticoagulants; Th-to-PrA: therapeutic and switching to prophylactic anticoagulants; ThA: therapeutic anticoagulants; VTE: venous thromboembolism ^a^ These analyses were for exploratory purposes, and no hypothesis testing was conducted. ^b^ PrA was the reference level for all statistical comparisons. ^c^ ORs were presented with a 95% confidence interval, which were calculated using g-estimation with bootstrapping. ^d^ Adjusted OR were controlled for age (years), sex (female or male), body mass index (kg/m2), chronic comorbidities (cardiovascular diseases, endocrine diseases, respiratory diseases, gastrointestinal diseases, or cancers), current smoker (yes or no), vaccination status (fully, partially, or no vaccination), biomarkers for treatment (CRP, D-dimer, and ferritin), and concurrent medications for COVID-19 treatment (corticosteroids, antivirals, antibiotics/antifungals). ^e^ The primary effectiveness outcome was a composite of all-cause mortality, ICU admission, stroke (either ischemic or hemorrhagic), and VTE. ^f^ These estimates were unreliable due to too few events being observed. ^g^ The primary safety outcome was a composite of major and minor/CRNM bleeding, defined using the classification of the International Society on Thrombosis and Haemostasis. ^h^ The net-benefit outcome was a composite of all-cause mortality, ICU admission, stroke, VTE, and major bleeding.

Outcomes^a^	PrA^b^ (n = 429)	Pr-to-ThA (n = 266)			Th-to-PrA (n = 114)			ThA (n = 179)		
	n (%)	n (%)	Unadjusted OR^c^	Adjusted OR^c,d^	n (%)	Unadjusted OR^c^	Adjusted OR^c,d^	n (%)	Unadjusted OR^c^	Adjusted OR^c,d^
Primary effectiveness outcome^e^	106 (24.7)	45 (16.9)	0.62 (0.42 to 0.91)	0.64 (0.46 to 0.88)	24 (21.1)	0.81 (0.49 to 1.34)	0.83 (0.51 to 1.30)	65 (36.3)	1.74 (1.19 to 2.53)	1.79 (1.22 to 2.57)
Secondary effectiveness outcomes										
All-cause mortality	105 (24.5)	40 (15.0)	0.55 (0.37 to 0.82)	0.57 (0.38 to 0.80)	12 (10.5)	0.36 (0.19 to 0.69)	0.37 (0.23 to 0.65)	46 (25.7)	1.07 (0.71 to 1.59)	1.29 (0.77 to 2.02)
ICU admission	101 (23.5)	43 (16.2)	0.63^f^ (0.42 to 0.93)	0.65 (0.45 to 0.91)	20 (17.5)	0.69 (0.41 to 1.18)	0.68 (0.39 to 1.15)	35 (19.6)	0.79 (0.51 to 1.22)	0.82 (0.52 to 1.20)
Stroke	1 (0.2)	1 (0.4)	– ^f^	– ^f^	0 (0.0)	– ^f^	– ^f^	1 (0.6)	– ^f^	– ^f^
VTE	29 (6.8)	16 (6.0)	0.88 (0.47 to 1.66)	0.89 (0.48 to 1.65)	6 (5.3)	0.77 (0.31 to 1.89)	0.88 (0.42 to 1.77)	9 (5.0)	0.73 (0.34 to 1.58)	0.67 (0.28 to 1.59)
Mechanical ventilation	68 (15.9)	39 (14.7)	0.91 (0.59 to 1.40)	0.93 (0.61 to 1.38)	17 (14.9)	0.93 (0.52 to 1.66)	1.01 (0.58 to 1.70)	19 (10.6)	0.63 (0.37 to 1.08)	0.96 (0.45 to 1.18)
Primary safety outcome^g^	35 (8.2)	22 (8.3)	1.01 (0.58 to 1.77)	1.03 (0.60 to 1.74)	19 (16.7)	2.25 (1.23 to 4.11)	2.65 (1.58 to 5.04)	24 (13.4)	1.74 (1.00 to 3.03)	2.48 (1.33 to 4.69)
Secondary safety outcomes										
Major bleeding	5 (1.2)	4 (1.5)	– ^f^	– ^f^	2 (1.8)	– ^f^	– ^f^	4 (2.2)	– ^f^	– ^f^
Minor/CRNM bleeding	30 (7.0)	18 (6.8)	0.97 (0.53 to 1.77)	1.01 (0.56 to 1.75)	17 (14.9)	2.33 (1.24 to 4.40)	2.59 (1.55 to 5.12)	20 (11.2)	1.67 (0.92 to 3.03)	1.54 (0.87 to 2.94)
Net-benefit outcome^h^	108 (25.2)	46 (17.3)	0.62 (0.42 to 0.91)	0.64 (0.45 to 0.87)	24 (21.1)	0.79 (0.48 to 1.31)	0.83 (0.49 to 1.35)	68 (38.0)	1.82 (1.25 to 2.64)	1.88 (1.29 to 2.66)

We observed the primary safety outcome in 104 patients (35/486 (7.2%) in PrA group, 22/293 (7.5%) in Pr-to-ThA group, 19/118 (16.1%) in Th-to-PrA group, and 28/184 (15.2%) in ThA group, Table 2). Compared with patients receiving PrA, the risks of bleeding were higher in the Th-to-PrA (adjusted OR = 3.00, 95% CI: 1.53 to 5.89) and ThA groups (adjusted OR = 3.05, 95% CI: 1.61 to 5.79) (Table 2). Our post-hoc analysis using the Dunnett test showed that with a family-wise error rate of 5%, statistically testing the hypotheses of increasing risks of bleeding in the Th-to-PrA and ThA groups gave the multiplicity-adjusted values of p = 0.004 and p = 0.002, respectively. However, as this analysis was not planned a priori with an overall family-wise error rate, these results were considered exploratory only. Associations between the covariates and the primary safety outcomes are presented in Appendix D.

Secondary outcomes

In comparison with PrA groups, multiplicity-unadjusted results suggested some associations between the Pr-to-ThA dosing and ICU admission (adjusted OR = 0.67, 95% CI: 0.47 to 0.95) and net-benefit outcome (adjusted OR = 0.65, 95% CI: 0.46 to 0.92) (Table 2). For the Th-to-PrA, the potentially associated secondary outcomes were all-cause mortality (adjusted OR=0.43, 95% CI: 0.23 to 0.82), ICU admission (adjusted OR=0.49, 95% CI: 0.28 to 0.84), minor/CRNM bleeding (adjusted OR=2.89, 95% CI: 1.43 to 5.85), and net-benefit outcome (adjusted OR=0.60, 95% CI: 0.36 to 1.00) (Table 2). Patients in the ThA group were less likely to be admitted to the ICU (adjusted OR=0.59, 95% CI: 0.37 to 0.94) but more likely to experience minor/CRNM bleeding (adjusted OR=2.83, 95% CI: 1.44 to 5.55) than those in the PrA group (Table 2). Among the four groups, there were no episodes of fatal bleeding or bleeding that required blood transfusion. Associations between the covariates and the secondary outcomes are reported in Appendix E.

Exploratory analysis

Table 4 summarizes the results of the exploratory causal mediation analysis. Switching doses of anticoagulants was likely associated with a lower risk of having the primary effectiveness outcome (in Pr-to-ThA group, adjusted estimate -0.018, 95% CI: -0.035 to -0.004; in Th-to-PrA, adjusted estimate -0.034, 95% CI: -0.062 to -0.011) and the net-benefit outcome (in Pr-to-ThA group, adjusted estimate -0.017, 95% CI: -0.033 to -0.003; in Th-to-PrA, adjusted estimate -0.035, 95% CI: -0.061 to -0.011) (Table 4). Within the switching groups, Pr-to-ThA dosing was associated with reduced risk of ICU admission (adjusted estimate -0.016, 95% CI: -0.032 to -0.002), while patients with Th-to-PrA dosing had a lower probability of mortality (adjusted estimate -0.037, 95% CI: -0.061 to -0.015) (Table 4). For patients with initial treatment of ThA, maintaining the therapeutic dose was likely associated with increasing bleeding risk (adjusted estimate 0.107, 95% CI: 0.046 to 0.177) (Table 4).

**Table 4:** Results of the exploratory model-based causal mediation analysis CRNM: clinically relevant non-major; ICU: intensive care unit; PrA: prophylactic anticoagulants; Pr-to-ThA: prophylactic and switching to therapeutic anticoagulants; Th-to-PrA: therapeutic and switching to prophylactic anticoagulants; ThA: therapeutic anticoagulants; VTE: venous thromboembolism ^a^ The estimates were presented on a probability scale with a 95% confidence interval. Negative signs implicated reversed associations of the exposure on the outcomes. Values of 0.000 or -0.000 indicated very small associations (lower than 0.001). ^b^ Adjusted estimates were controlled for age (years), sex (female or male), body mass index (kg/m^2^), chronic comorbidities (cardiovascular diseases, endocrine diseases, respiratory diseases, gastrointestinal diseases, or cancers), current smoker (yes or no), vaccination status (fully, partially, or no vaccination), biomarkers for treatment (CRP, D-dimer, and ferritin), and concurrent medications for COVID-19 treatment (corticosteroids, antivirals, antibiotics/antifungals). ^c^ The primary effectiveness outcome was a composite of all-cause mortality, ICU admission, stroke (either ischemic or hemorrhagic), and VTE. ^d^ All outcomes in the causal mediation analysis were considered exploratory. ^e^ These estimates were unreliable due to too few events being observed. ^f^ The primary safety outcome was a composite of major and minor/CRNM bleeding, defined using the classification of the International Society on Thrombosis and Haemostasis. ^g^ The net-benefit outcome was a composite of all-cause mortality, ICU admission, stroke, and major bleeding.

Outcomes	PrA		Pr-to-ThA		Th-to-PrA		ThA	
	Unadjusted estimate^a^	Adjusted estimate^a,b^	Unadjusted estimate^a^	Adjusted estimate^a,b^	Unadjusted estimate^a^	Adjusted estimate^a,b^	Unadjusted estimate^a^	Adjusted estimate^a,b^
Primary effectiveness outcome^c,d^	-0.010 (-0.069 to 0.057)	-0.014 (-0.085 to 0.062)	-0.020 (-0.037 to -0.006)	-0.018 (-0.035 to -0.004)	-0.033 (-0.057 to -0.009)	-0.034 (-0.062 to -0.011)	0.043 (-0.014 to 0.106)	0.038 (-0.036 to 0.115)
Secondary effectiveness outcomes^d^								
All-cause mortality	-0.028 (-0.080 to 0.031)	-0.025 (-0.087 to 0.042)	-0.014 (-0.028 to 0.000)	-0.011 (-0.025 to 0.002)	-0.032 (-0.057 to -0.010)	-0.037 (-0.061 to -0.015)	0.018 (-0.037 to 0.080)	0.023 (-0.042 to 0.089)
ICU admission	-0.085 (-0.143 to -0.024)	-0.082 (-0.147 to -0.011)	-0.018 (-0.034 to -0.003)	-0.016 (-0.032 to -0.002)	0.003 (-0.020 to 0.023)	0.007 (-0.013 to 0.028)	-0.063 (-0.118 to -0.005)	-0.059 (-0.124 to 0.008)
Stroke	– ^e^	– ^e^	– ^e^	– ^e^	– ^e^	– ^e^	– ^e^	– ^e^
VTE	– ^e^	– ^e^	– ^e^	– ^e^	– ^e^	– ^e^	– ^e^	– ^e^
Mechanical ventilation	0.006 (-0.043 to 0.061)	0.041 (-0.018 to 0.104)	-0.002 (-0.016 to 0.010)	-0.002 (-0.013 to 0.009)	-0.012 (-0.033 to 0.006)	-0.012 (-0.037 to 0.008)	-0.004 (-0.052 to 0.042)	0.030 (-0.026 to 0.087)
Primary safety outcome^d,f^	-0.085 (-0.141 to -0.038)	-0.109 (-0.182 to -0.046)	0.001 (-0.008 to 0.010)	0.001 (-0.008 to 0.011)	-0.002 (-0.022 to 0.017)	-0.000 (-0.020 to 0.020)	0.082 (0.037 to 0.130)	0.107 (0.046 to 0.177)
Secondary safety outcomes^d^								
Major bleeding	– ^e^	– ^e^	– ^e^	– ^e^	– ^e^	– ^e^	– ^e^	– ^e^
Minor/CRNM bleeding	-0.079 (-0.128 to -0.032)	-0.093 (-0.162 to -0.040)	0.000 (-0.007 to 0.009)	0.000 (-0.008 to 0.009)	-0.003 (-0.024 to 0.014)	-0.001 (-0.022 to 0.019)	0.075 (0.031 to 0.119)	0.092 (0.035 to 0.156)
Net-benefit outcome^d,g^	-0.006 (-0.063 to 0.059)	0.001 (-0.070 to 0.071)	-0.020 (-0.038 to -0.004)	-0.017 (-0.033 to -0.003)	-0.035 (-0.059 to -0.011)	-0.035 (-0.061 to -0.011)	0.048 (-0.012 to 0.111)	0.054 (-0.017 to 0.123)

## Discussion

Overall, the Pr-to-ThA dosing was associated with a significantly lower risk of all-cause mortality, ICU admission, stroke, or VTE than the standard PrA dosing over a median follow-up of 10 days. The risks of major or minor/CRNM bleeding were probably higher in patients receiving Th-to-PrA and ThA compared with the PrA group. After disentangling the associations of the initial and final dosing from the overall estimates, we found that switching the dose of anticoagulants was likely associated with a lower risk of all-cause mortality, ICU admission, stroke, or VTE, whereas ThA dosing was associated with an increase in the bleeding risk.

The effectiveness profile of the ThA dosing was inconsistent with findings from prior publications [8,10,11]. This was probably because many patients in the ThA group had persistently elevated biomarkers for VTE and severe illness, which were prognostic factors for poor outcomes, i.e., mortality or ICU admission [19], whereas most included patients in other studies were moderately ill or not requiring respiratory support [8,10]. During this wave of COVID-19 in Vietnam, we did not have enough supply to closely monitor the anti-Xa activity in all patients on ThA therapy. Instead, we had to use the activated partial thromboplastin time assay, which was not recommended for monitoring ThA in patients with COVID-19 [20]. This could partially explain the increased risk of bleeding in this cohort, unlike the safety profile of ThA from other studies [8-11].

While Pr-to-ThA dosing was associated with a lower risk of the primary effectiveness outcome, this result was primarily driven by a lower risk of ICU admission. The causal mediation analysis also provided evidence supporting this separate association of dose-increasing anticoagulants. This may imply the benefits of using a higher dose of anticoagulants during the treatment course, which partially supported the new practice of fixed therapeutic dosing for patients with moderate-to-severe COVID-19 [1,4,21,22]. The risk of major or minor/CRNM bleeding was comparable between the Pr-to-ThA and the standard PrA group, which showed a similar safety profile compared to prior reports [8-11].

For the Th-to-PrA dosing, with the current sample size, we could not detect a significant reduction in the composite risk of all-cause mortality, ICU admission, stroke, or VTE. The causal mediation analysis implicated that with a larger sample size, we might find an association with a lower risk of mortality, which could potentially show a significant benefit of the Th-to-PrA dosing. In that case, our findings would be consistent with evidence from previous trials [8,10,11], thus strongly supporting the practice of initiating ThA in patients with elevated levels of biomarkers for VTE [1,4]. Although patients on Th-to-PrA were more likely to experience minor/CRNM bleeding events, the safety profile in major bleeding of the Th-to-PrA dosing was similar to what has been reported [8-11].

Our findings implied that step-based dosing of anticoagulants could be a prognostic factor for patients with moderate-to-severe COVID-19. In cases of COVID-19 outbreaks and resource scarcity, patients at higher risk (e.g., receiving ThA) could be offered more care and monitoring to optimize treatment outcomes. Hospitals from low-middle-income countries may implement this practice to guide resource allocation during difficult times. Additionally, this step-based dosing strategy is also applicable to conditions with similar dynamic changes throughout the treatment course. However, evidence in these settings is still needed before implementing this practice.

This study had several limitations. First, we did not have specific information about the use of oral anticoagulants before hospitalization, as most patients did not recall which medications they had been taking at home for COVID-19 symptoms. As oral anticoagulants were strictly reserved for patients who required hospitalization but were delayed due to healthcare overload, only a small proportion of patients was estimated to have used these medications before admission. Second, as data were collected from a single site only, these findings may not be generalizable to other settings. Third, we did not include data on patients with critical illness, as we could not control for other prognostic factors in this subpopulation. Fourth, we only considered one time point for the dose-switching groups, while the actual step-based dosing of anticoagulants may require more frequent changes. Fifth, the sample sizes for the ThA-to-PrA and ThA cohorts may not be adequate to detect all the significant differences in the outcomes. Finally, we did not have specific data about the pathogenic variants of all included patients, but based on local statistics, Delta was the dominant variant during this wave of COVID-19.

## Conclusions

Compared with the standard PrA, the step-based dose-increasing therapy was associated with a lower composite risk of all-cause mortality, ICU admission, stroke, or VTE without evidence of a higher risk of bleeding in patients with moderate-to-severe COVID-19. Dosing ofThA was associated with an increase in the bleeding risk, primarily minor and CRNM bleeding. There might be some benefits in mortality for the step-based dose-decreasing anticoagulants, but more studies are needed to confirm this hypothesis.

## Appendices

Appendix A

eMethods: Design, Setting, and Participants

Inclusion criteria: (1) were ≥18 years old; (2) got confirmed COVID-19 (COVID-19 diagnosis was verified by either a positive rapid antigen test with typical symptoms or a positive real-time polymerase chain reaction test); (3) were hospitalized at Nhan Dan Gia Dinh Hospital during the period between August 1, 2021, and November 30, 2021; (4) had moderate-to-severe illness during admission (patients with moderate illness were individuals who showed evidence of lower respiratory disease during clinical assessment or imaging and who have an oxygen saturation measured by pulse oximetry (SpO2) ≥94% on room air at sea level; patients with severe illness were individuals who have SpO2 <94% on room air at sea level, a ratio of arterial partial pressure of oxygen to fraction of inspired oxygen (PaO2/FiO2) <300 mmHg, a respiratory rate >30 breaths/min, or pulmonary infiltrates >50%); and (5) received at least 1 dose of heparin-based anticoagulants, i.e., unfractionated heparin or low-molecular-weight heparin.

Exclusion criteria: (1) were pregnant or breastfeeding; (2) were moderately or severely immunocompromised (white blood cell count <4×109/L, active cancer treatment, immunosuppressive medications, moderate or severe primary immunodeficiency, advanced or untreated human immunodeficiency virus infection); (3) had renal impairment (creatinine clearance, CrCl, <30 mL/min); (4) had hepatic impairment (Child-Pugh class C); or (5) required therapeutic anticoagulation or dual antiplatelet therapy for underlying conditions.

Exposure

The exposure of interest in this study was the clinical step-based dosing of anticoagulants. This divided the study sample into 4 cohorts, of which patients were given: (1) prophylactic anticoagulants (PrA); (2) prophylactic and later switching to therapeutic anticoagulants (Pr-to-ThA); (3) therapeutic anticoagulants (ThA); and (4) therapeutic and later switching to prophylactic anticoagulants (Th-to-PrA). The timing for switching in the Pr-to-ThA and Th-to-PrA groups could be anytime between day 1 (first dose) and day 10. If there was a switch after day 10, patients were classified based only on their first doses of anticoagulants, which were either PrA or ThA.

Definitions of anticoagulant dosing were based on patients’ CrCl and body mass index (BMI) as follows (IV: intravenous, SC: subcutaneous). PrA (CrCl ≥30 mL/min): (1) BMI ≤30kg/m2: heparin 5000 IU (SC), q12h; or enoxaparin 4000 IU (SC), q24h; (2) BMI >30kg/m2: heparin 7500 IU (SC), q12h; or enoxaparin 4000 IU (SC), q12h-q24h. ThA (CrCl ≥30 mL/min): (1) BMI ≤30kg/m2: heparin 5000 IU or 80 IU/kg (IV bolus), then 250 IU/kg q12h or 18 IU/kg/hr (SC); or enoxaparin 100 IU/kg (SC), q12h; (2) BMI >30kg/m2: enoxaparin 80 IU/kg (SC), q12h. For reference purposes, dosing approaches for patients with CrCl <30 mL/min, who were excluded from the study, were as follows: (1) PrA: heparin 5000-7000 IU (SC), q8h-q12h; or enoxaparin 3000 IU (SC), q24h; (2) ThA: heparin 5000 IU or 80 IU/kg (IV bolus), then 250 IU/kg q12h or 18 IU/kg q1h (SC); or enoxaparin 100 IU/kg (SC), q24h.

The dosing strategies of anticoagulants were decided depending on the risk of VTE, which followed a step-based national protocol. This protocol used the following biomarkers/signs to guide anticoagulant dosing: C-reactive protein (CRP), ferritin, D-dimer, interleukin-6 (IL-6), and pulmonary infiltrates. These laboratory tests were indicated at baseline and after an average of every 2 or 3 days. If there were concerning symptoms, certain biomarkers could be indicated immediately.

Patients were given PrA if: (1) CRP ≤15 mg/L; (2) ferritin ≤1000 ng/mL; (3) D-dimer ≥2 and <5 times the upper limit of normal; (4) IL-6 15-40 pg/mL; and (5) no pulmonary infiltrates. Patients were given ThA if: (1) CRP >15 mg/L; (2) ferritin >1000 ng/mL; or (3) D-dimer ≥5 times the upper limit of normal or doubled within 24-48 hours; (4) IL-6 >40 pg/mL; or (5) presence of pulmonary infiltrates. In our study setting, as new evidence of VTE in COVID-19 emerged rapidly, different physicians had different approaches for dosing anticoagulants. During the COVID-19 outbreaks, adherence to the protocols was not strictly imposed at our hospital. Thus, many physicians did not comply with the national protocol, resulting in some variations of the dosing strategies. For instance, patients initially receiving PrA might not get switched to ThA even when they met the criteria of the national protocol if the physicians did not anticipate any benefits of dose escalation.

Appendix B

**Table 5:** STROBE statement: checklist of items that should be included in reports of cohort studies. ^a^ Give information separately for exposed and unexposed groups. Note: An explanation and elaboration article discusses each checklist item and gives methodological background and published examples of transparent reporting. The STROBE checklist is best used in conjunction with this article (freely available on the Web sites of PLoS Medicine at http://www.plosmedicine.org/, Annals of Internal Medicine at http://www.annals.org/, and Epidemiology at http://www.epidem.com/). Information on the STROBE Initiative is available at http://www.strobe-statement.org. STROBE: Strengthening the Reporting of Observational Studies in Epidemiology

	Item No	Recommendation
Title and abstract		
	1	Explain the scientific background and rationale for the investigation being reported Introduction (paragraph 1 and 2)
		State specific objectives, including any prespecified hypotheses Introduction (paragraph 3)
Introduction		
Background/rationale	2	Explain the scientific background and rationale for the investigation being reported Introduction (paragraph 1 and 2)
Objectives	3	State specific objectives, including any prespecified hypotheses Introduction (paragraph 3)
Methods		
Study design	4	Present key elements of study design early in the paper Methods (Design, Setting, and Participants)
Setting	5	Describe the setting, locations, and relevant dates, including periods of recruitment, exposure, follow-up, and data collection Methods (Design, Setting, and Participants)
Participants	6	(a) Give the eligibility criteria, and the sources and methods of selection of participants. Describe methods of follow-up Methods (Design, Setting, and Participants)
		(b) For matched studies, give matching criteria and number of exposed and unexposed NA
Variables	7	Clearly define all outcomes, exposures, predictors, potential confounders, and effect modifiers. Give diagnostic criteria, if applicable Methods (Exposure; Outcomes; Covariates)
Data sources/ management	8^a^	For each variable of interest, give sources of data and details of methods of assessment (measurement). Describe comparability of assessment methods if there is more than one group Methods (Exposure; Outcomes)
BiasParticipants	9	Describe any efforts to address potential sources of bias Methods (Outcomes; Covariates; Statistical Analysis)
Study size	10	Explain how the study size was arrived at Methods (Statistical Analysis)
Quantitative variables	11	Explain how quantitative variables were handled in the analyses. If applicable, describe which groupings were chosen and why Methods (Statistical Analysis)
Statistical methods	12	(a) Describe all statistical methods, including those used to control for confounding Methods (Statistical Analysis)
		(b) Describe any methods used to examine subgroups and interactions Methods (Statistical Analysis)
		(c) Explain how missing data were addressed Methods (Statistical Analysis)
		(d) If applicable, explain how loss to follow-up was addressed Methods (Statistical Analysis)
		(e) Describe any sensitivity analyses NA
Results		
Participants	13*	(a) Report numbers of individuals at each stage of study—eg numbers potentially eligible, examined for eligibility, confirmed eligible, included in the study, completing follow-up, and analysed Results (Figure 1)
		(b) Give reasons for non-participation at each stage Results (Figure 1)
		(c) Consider use of a flow diagram Results (Figure 1)
Descriptive data	14*	(a) Give characteristics of study participants (eg demographic, clinical, social) and information on exposures and potential confounders Results (Table 1; Baseline Characteristics)
		(b) Indicate number of participants with missing data for each variable of interest Results (Figure 1)
		(c) Summarise follow-up time (eg, average and total amount) Results (Primary Outcomes)
Outcome data	15*	Report numbers of outcome events or summary measures over time Results (Table 2)
Main results	16	(a) Give unadjusted estimates and, if applicable, confounder-adjusted estimates and their precision (eg, 95% confidence interval). Make clear which confounders were adjusted for and why they were included Results (Table 2; Primary Outcomes; Secondary Outcomes)
		(b) Report category boundaries when continuous variables were categorized Results (Figure 2)
		(c) If relevant, consider translating estimates of relative risk into absolute risk for a meaningful time period NA
Other analyses	17	Report other analyses done—eg analyses of subgroups and interactions, and sensitivity analyses Results (Table 3; Exploratory Analysis)
Discussion		
Key results	18	Summarise key results with reference to study objectives Discussion (paragraph 1)
Limitations	19	Discuss limitations of the study, taking into account sources of potential bias or imprecision. Discuss both direction and magnitude of any potential bias Discussion (Limitations)
Interpretation	20	Give a cautious overall interpretation of results considering objectives, limitations, multiplicity of analyses, results from similar studies, and other relevant evidence Discussion (paragraph 2, 3, 4)
Generalisability	21	Discuss the generalisability (external validity) of the study results Discussion (paragraph 2, 3, 4)
Other information		
Funding	22	Give the source of funding and the role of the funders for the present study and, if applicable, for the original study on which the present article is based NA

Appendix C 

**Table 6:** Patient characteristics of the included and excluded medical records. BMI: body mass index; CRP: C-reactive protein; IQR: interquartile range; PrA: prophylactic anticoagulants; Pr-to-ThA: prophylactic and switching to therapeutic anticoagulants; Th-to-PrA: therapeutic and switching to prophylactic anticoagulants; ThA: therapeutic anticoagulants. ^a^ p-values were calculated using Fisher test for categorical variables or Mann–Whitney test for continuous variables. ^b^ Percentages may not equate to 100 due to concurrent status or rounding. ^c^ Current smoker refers to an adult who has smoked ≥100 cigarettes in their lifetime and who currently smokes cigarettes. ^d^ Fully vaccinated persons were those who received at least 2 doses (either homologous or heterologous) of the approved vaccines for at least 2 weeks before getting the first COVID-19. Partially vaccinated persons were those who received only 1 dose before getting the first COVID-19. The approved vaccines in Vietnam included BNT162b2 (Pfizer/BioNTech), mRNA-1273 (Moderna), AZD1222 (AstraZeneca), and BBIBP-CorV (Sinopharm). ^e^ Corticosteroids included dexamethasone, methylprednisolone, and prednisolone. ^f^ Antivirals for COVID-19 treatment included remdesivir, molnupiravir, and favipiravir. ^g^ Antibiotics or antifungals were prescribed for patients with confirmed secondary infections. Treatments with these medications could be empirical and/or based on the susceptibility of the isolated pathogens.

Characteristics	PrA			Pr-to-ThA			Th-to-PrA			ThA		
	Included	Excluded	p^a^	Included	Excluded	p^a^	Included	Excluded	p^a^	Included	Excluded	p^a^
	(n=486)	(n=17)		(n=293)	(n=18)		(n=118)	(n=6)		(n=184)	(n=14)	
Age (years), median (IQR)	62 (50–70)	59 (42–68)	0.53	62 (48–69)	64 (59–68)	0.70	61 (51–70)	63 (51–66)	0.88	59 (51–69)	57 (46–71)	0.81
Female sex, n (%)	254 (52.3)	9 (52.9)	>0.99	144 (49.1)	9 (50.0)	>0.99	46 (39.0)	2 (33.3)	>0.99	95 (51.6)	8 (57.1)	0.79
BMI (kg/m^2^), median (IQR)	22.8 (21.3–25.1)	22.9 (21.4–24.2)	0.92	23.1 (21.4–25.3)	22.3 (21.2–24.1)	0.47	23.4 (21.5–27.1)	22.7 (22.1–23.5)	0.87	23.7 (21.5–26.7)	24.3 (21.4–26.5)	0.92
Chronic comorbidities, n (%)^b^												
Cancers	31 (6.4)	1 (5.9)	>0.99	22 (7.5)	2 (11.1)	0.68	3 (2.5)	0 (0.0)	>0.99	7 (3.8)	0 (0.0)	>0.99
Cardiovascular diseases	230 (47.3)	7 (41.2)	0.81	137 (46.8)	12 (66.7)	0.14	70 (59.3)	2 (33.3)	0.24	103 (56.0)	7 (50.0)	0.78
Endocrine diseases	159 (32.7)	5 (29.4)	>0.99	99 (33.8)	3 (16.7)	0.20	53 (44.9)	2 (33.3)	0.69	80 (43.5)	8 (57.1)	0.41
Gastrointestinal diseases	36 (7.4)	2 (11.8)	0.37	23 (7.8)	2 (11.1)	0.65	17 (14.4)	1 (16.7)	>0.99	25 (13.6)	1 (7.1)	0.70
Respiratory diseases	32 (6.6)	3 (17.6)	0.11	16 (5.5)	1 (5.6)	>0.99	8 (6.8)	1 (16.7)	0.37	11 (6.0)	2 (14.3)	0.23
Current smoker, n (%)^c^	72 (14.8)	0 (0.0)	0.15	55 (18.8)	4 (22.2)	0.76	20 (16.9)	2 (33.3)	0.29	32 (17.4)	4 (28.6)	0.29
Vaccination status, n (%)^b,d^			0.82			0.46			0.88			0.45
Fully	188 (38.7)	7 (41.2)		115 (39.2)	7 (38.9)		37 (31.4)	2 (33.3)		61 (33.2)	7 (50.0)	
Partially	147 (30.2)	6 (35.3)		98 (33.4)	4 (22.2)		34 (28.8)	1 (16.7)		58 (31.5)	3 (21.4)	
None	151 (31.1)	4 (23.5)		80 (27.3)	7 (38.9)		47 (39.8)	3 (50.0)		65 (35.3)	4 (28.6)	
Biomarkers, median (IQR)												
CRP (mg/L)	11.4 (6.3–13.7)	12.4 (7.1–13.0)	0.87	10.9 (6.8–12.1)	9.4 (6.2–13.3)	0.80	71.2 (44.5–92.1)	67.3 (51.2–88.9)	0.59	77.8 (32.9–118.4)	76.4 (46.1–91.7)	0.65
D-dimer (ng/mL)	1325 (1184–1790)	1208 (1095–1622)	0.79	1547 (1338–1859)	1393 (1140–1852)	0.54	2162 (1581–2736)	2238 (1496–2718)	0.68	2377 (1613–2710)	2207 (1558–2641)	0.61
Ferritin (ng/mL)	682 (536–870)	742 (681–893)	0.75	588 (479–812)	605 (491–795)	0.89	1268 (692–1830)	1316 (725–1782)	0.70	1356 (775–2025)	1410 (728–1982)	0.60
Concurrent medications, n (%)^b^												
Corticosteroids^e^	470 (96.7)	17 (100.0)	>0.99	284 (96.9)	18 (100.0)	>0.99	105 (89.0)	5 (83.3)	0.52	176 (95.7)	13 (92.9)	0.49
Antivirals^f^	105 (21.6)	3 (17.6)	>0.99	82 (28.0)	5 (27.8)	>0.99	36 (30.5)	2 (33.3)	>0.99	64 (34.8)	6 (42.9)	0.57
Antibiotics/antifungals^g^	146 (30.0)	7 (41.2)	0.42	75 (25.6)	2 (11.1)	0.26	85 (72.0)	4 (66.7)	>0.99	164 (89.1)	12 (85.7)	0.66

Appendix D 

**Table 7:** Associations between the covariates and the primary effectiveness and safety outcomes. BMI: body mass index; CRP: C-reactive protein ^a^ Estimates were presented as odds ratio with the 95% confidence intervals, which were calculated using g-estimation with bootstrapping. ^b^ The primary effectiveness outcome was a composite of all-cause mortality, ICU admission, and stroke (either ischemic or hemorrhagic). ^c^ The primary safety outcome was a composite of major and minor bleeding, defined using the classification of the International Society on Thrombosis and Haemostasis. ^d^ Current smoker refers to an adult who has smoked ≥100 cigarettes in their lifetime and who currently smokes cigarettes. ^e^ Fully vaccinated persons were those who received at least 2 doses (either homologous or heterologous) of the approved vaccines for at least 2 weeks before getting the first COVID-19. Partially vaccinated persons were those who received only 1 dose before getting the first COVID-19. The approved vaccines in Vietnam included BNT162b2 (Pfizer/BioNTech), mRNA-1273 (Moderna), AZD1222 (AstraZeneca), and BBIBP-CorV (Sinopharm). ^f^ Corticosteroids included dexamethasone, methylprednisolone, and prednisolone. ^g^ Antivirals for COVID-19 treatment included remdesivir, molnupiravir, and favipiravir. ^h^ Antibiotics or antifungals were prescribed for patients with confirmed secondary infections. Treatments with these medications could be empirical and/or based on the susceptibility of the isolated pathogens.

Covariates	Primary effectiveness outcome^a,b^	Primary safety outcome^a,c^
Age	1.04 [1.03, 1.05]	1.03 [1.01, 1.05]
Female sex	0.93 [0.70, 1.23]	1.13 [0.73, 1.74]
BMI	1.05 [1.01 to 1.08]	0.96 [0.90, 1.02]
Chronic comorbidities		
Cancers	1.22 [0.69, 2.15]	1.17 [0.51, 2.68]
Cardiovascular diseases	0.80 [0.59, 1.10]	1.45 [0.90, 2.35]
Endocrine diseases	1.19 [0.88, 1.60]	0.84 [0.54, 1.33]
Gastrointestinal diseases	0.74 [0.46, 1.21]	4.46 [1.57, 12.66]
Respiratory diseases	1.15 [0.56, 2.36]	1.05 [0.60, 1.82]
Current smoker^d^	1.01 [0.69, 1.48]	1.23 [0.70, 2.15]
Vaccination statute		
Fully	reference	reference
Partially	0.71 [0.50, 1.01]	1.03 [0.59, 1.81]
None	1.95 [1.41, 2.70]	1.72 [1.03, 2.86]
Log-transformed biomarkers		
CRP	1.12 [0.83, 1.52]	1.06 [0.79, 1.42]
D-dimer	1.18 [0.96, 1.45]	1.07 [0.76, 1.51]
Ferritin	1.11 [0.87, 1.42]	1.01 [0.69, 1.48]
Concurrent medications		
Corticosteroids^f^	0.47 [0.25, 0.89]	0.72 [0.28, 1.83]
Antivirals^g^	0.77 [0.55, 1.07]	1.18 [0.73, 1.90]
Antibiotics/antifungals^h^	1.00 [0.72, 1.39]	0.77 [0.46, 1.29]

Appendix E 

**Table 8:** Associations between the covariates and the secondary outcomes. BMI: body mass index; CRP: C-reactive protein; MV: mechanical ventilation. ^a^ Estimates were presented as odds ratio with the 95% confidence intervals, which were calculated using g-estimation with bootstrapping. ^b^ These estimates were unreliable due to too few events being observed. ^c^ Current smoker refers to an adult who has smoked ≥100 cigarettes in their lifetime and who currently smokes cigarettes. ^d^ Fully vaccinated persons were those who received at least 2 doses (either homologous or heterologous) of the approved vaccines for at least 2 weeks before getting the first COVID-19. Partially vaccinated persons were those who received only 1 dose before getting the first COVID-19. The approved vaccines in Vietnam included BNT162b2 (Pfizer/BioNTech), mRNA-1273 (Moderna), AZD1222 (AstraZeneca), and BBIBP-CorV (Sinopharm). ^e^ Corticosteroids included dexamethasone, methylprednisolone, and prednisolone. ^f^ Antivirals for COVID-19 treatment included remdesivir, molnupiravir, and favipiravir. ^g^ Antibiotics or antifungals were prescribed for patients with confirmed secondary infections. Treatments with these medications could be empirical and/or based on the susceptibility of the isolated pathogens.

Covariates	Secondary effectiveness outcomes^a^					Secondary safety outcomes^a^		Net-benefit outcome^a^
	All-cause mortality	ICU admission	Stroke	VTE	MV	Major bleeding	Minor bleeding	
Age	1.05 [1.04, 1.06]	1.04 [1.03, 1.05]	–^ b^	1.04 [1.02, 1.07]	1.06 [1.04, 1.08]	–^ b^	1.02 [1.00, 1.04]	1.04 [1.03, 1.05]
Female sex	0.96 [0.70, 1.33]	0.86 [0.64, 1.16]	–^ b^	0.81 [0.46, 1.40]	1.30 [0.90, 1.87]	–^ b^	1.05 [0.66, 1.66]	0.95 [0.72, 1.26]
BMI	1.04 [1.01, 1.08]	1.07 [1.03, 1.11]	–^ b^	1.04 [0.99, 1.09]	1.05 [1.01, 1.09]	–^ b^	0.95 [0.89, 1.02]	1.05 [1.02, 1.09]
Chronic comorbidities								
Cancers	0.91 [0.49, 1.72]	1.29 [0.72, 2.31]	–^ b^	0.46 [0.10, 2.00]	2.24 [1.22, 4.12]	–^ b^	1.23 [0.52, 2.94]	1.25 [0.71, 2.20]
Cardiovascular diseases	0.90 [0.64, 1.28]	0.86 [0.62, 1.19]	–^ b^	0.79 [0.43, 1.43]	1.08 [0.72, 1.60]	–^ b^	1.33 [0.80, 2.20]	0.82 [0.60, 1.12]
Endocrine diseases	1.16 [0.83, 1.62]	0.93 [0.68, 1.27]	–^ b^	–^ b^	0.88 [0.60, 1.28]	–^ b^	1.04 [0.65, 1.68]	1.07 [0.79, 1.44]
Gastrointestinal diseases	0.80 [0.47, 1.36]	0.61 [0.36, 1.05]	–^ b^	0.86 [0.32, 2.33]	0.57 [0.30, 1.07]	–^ b^	1.01 [0.61, 1.70]	0.70 [0.43, 1.13]
Respiratory diseases	0.57 [0.28, 1.15]	1.10 [0.53, 2.32]	–^ b^	0.68 [0.20, 2.27]	0.97 [0.48, 1.95]	–^ b^	0.29 [0.07, 1.24]	1.21 [0.59, 2.49]
Current smoker^c^	0.90 [0.57, 1.41]	0.94 [0.63, 1.41]	–^ b^	0.85 [0.40, 1.82]	0.94 [0.57, 1.56]	–^ b^	1.08 [0.59, 1.99]	1.07 [0.73, 1.56]
Vaccination status^d^								
Fully	reference	reference	–^ b^	reference	reference	–^ b^	reference	reference
Partially	0.78 [0.51, 1.19]	0.86 [0.61, 1.20]	–^ b^	0.49 [0.24, 1.03]	1.02 [0.64, 1.61]	–^ b^	1.09 [0.60, 1.96]	0.71 [0.50, 1.01]
None	2.55 [1.77, 3.68]	2.03 [1.45, 2.83]	–^ b^	1.05 [0.57, 1.91]	1.75 [1.14, 2.68]	–^ b^	1.59 [0.93, 2.74]	1.94 [1.41, 2.68]
Log-transformed biomarkers								
CRP	1.18 [0.90, 1.56]	1.08 [0.84, 1.39]	–^ b^	1.20 [0.93, 1.55]	2.39 [1.72, 3.24]	–^ b^	1.05 [0.77, 1.43]	1.13 [0.85, 1.50]
D-dimer	1.14 [0.89, 1.46]	1.10 [0.81, 1.49]	–^ b^	1.26 [1.08, 1.47]	1.87 [1.49, 2.35]	–^ b^	1.03 [0.73, 1.45]	1.19 [0.94, 1.50]
Ferritin	1.09 [0.78, 1.52]	1.11 [0.82, 1.50]	–^ b^	1.17 [0.97, 1.41]	1.38 [0.94, 2.03]	–^ b^	0.99 [0.67, 1.47]	1.13 [0.89, 1.44]
Concurrent medications								
Corticosteroids^e^	0.84 [0.38, 1.87]	0.46 [0.24, 0.91]	–^ b^	–^ b^	2.77 [0.79, 9.76]	–^ b^	0.62 [0.24, 1.57]	0.44 [0.23, 0.83]
Antivirals^f^	0.50 [0.33, 0.75]	0.64 [0.44, 0.93]	–^ b^	1.56 [0.86, 2.85]	1.28 [0.85, 1.94]	–^ b^	0.28 [0.10, 0.80]	0.80 [0.58, 1.11]
Antibiotics/antifungals^g^	0.92 [0.63, 1.34]	0.96 [0.69, 1.34]	–^ b^	1.50 [0.82, 2.76]	0.75 [0.49, 1.15]	–^ b^	0.81 [0.47, 1.40]	0.96 [0.69, 1.33]
